# AeMOPE-1, a Novel Salivary Peptide From *Aedes aegypti*, Selectively Modulates Activation of Murine Macrophages and Ameliorates Experimental Colitis

**DOI:** 10.3389/fimmu.2021.681671

**Published:** 2021-07-19

**Authors:** Priscila G. Lara, Eliane Esteves, Helioswilton Sales-Campos, Josiane B. Assis, Maressa O. Henrique, Michele S. Barros, Leila S. Neto, Pedro I. Silva, Joilson O. Martins, Cristina R. B. Cardoso, José M. C. Ribeiro, Anderson Sá-Nunes

**Affiliations:** ^1^ Department of Immunology, Institute of Biomedical Sciences, University of Sao Paulo, Sao Paulo, Brazil; ^2^ Department of Clinical Analysis, Toxicology and Food Sciences, School of Pharmaceutical Sciences of Ribeirao Preto, University of Sao Paulo, Ribeirao Preto, Brazil; ^3^ Laboratory for Applied Toxinology, Butantan Institute, Sao Paulo, Brazil; ^4^ Department of Clinical and Toxicological Analyses, School of Pharmaceutical Sciences, University of Sao Paulo, Sao Paulo, Brazil; ^5^ Section of Vector Biology, Laboratory of Malaria and Vector Research, National Institute of Allergy and Infectious Diseases, National Institutes of Health, Rockville, MD, United States; ^6^ National Institute of Science and Technology in Molecular Entomology, National Council of Scientific and Technological Development (INCT-EM/CNPq), Rio de Janeiro, Brazil

**Keywords:** *Aedes aegypti*, AeMOPE-1, macrophages, immunomodulation, experimental colitis

## Abstract

The sialotranscriptomes of *Aedes aegypti* revealed a transcript overexpressed in female salivary glands that codes a mature 7.8 kDa peptide. The peptide, specific to the *Aedes* genus, has a unique sequence, presents a putative secretory nature and its function is unknown. Here, we confirmed that the peptide is highly expressed in the salivary glands of female mosquitoes when compared to the salivary glands of males, and its secretion in mosquito saliva is able to sensitize the vertebrate host by inducing the production of specific antibodies. The synthetic version of the peptide downmodulated nitric oxide production by activated peritoneal murine macrophages. The fractionation of a *Ae. aegypti* salivary preparation revealed that the fractions containing the naturally secreted peptide reproduced the nitric oxide downmodulation. The synthetic peptide also selectively interfered with cytokine production by murine macrophages, inhibiting the production of IL-6, IL-12p40 and CCL2 without affecting TNF-α or IL-10 production. Likewise, intracellular proteins associated with macrophage activation were also distinctively modulated: while iNOS and NF-κB p65 expression were diminished, IκBα and p38 MAPK expression did not change in the presence of the peptide. The anti-inflammatory properties of the synthetic peptide were tested *in vivo* on a dextran sulfate sodium-induced colitis model. The therapeutic administration of the *Ae. aegypti* peptide reduced the leukocytosis, macrophage activity and nitric oxide levels in the gut, as well as the expression of cytokines associated with the disease, resulting in amelioration of its clinical signs. Given its biological properties *in vitro* and *in vivo*, the molecule was termed ***Ae*** *des*-specific **MO**dulatory **PE**ptide (AeMOPE-1). Thus, AeMOPE-1 is a novel mosquito-derived immunobiologic with potential to treat immune-mediated disorders.

## Introduction

In anautogenous mosquito species such as *Aedes aegypti* (Linnaeus, 1762), the adult females are required to feed on blood to trigger the release of neurohormones that stimulate the ovaries to produce ecdysteroidogenic hormone and, consequently, induce the fat body to produce vitellogenin and other yolk proteins used in egg formation (1–3). A successful blood feeding depends on the ability to counteract the host hemostatic, inflammatory and immune barriers, and mosquito saliva is the vital element to achieve this goal due to the pharmacological activities of its components. In the last 30 years, a devoted effort of many research groups unveiled the roles of *Ae. aegypti* saliva and its isolated molecules on platelet aggregation (4–6), vasoconstriction (7, 8) and blood coagulation (9, 10). On the other hand, despite advances on the immunological basis of allergic reactions to *Ae. aegypti* salivary proteins (11–13), the knowledge on the biological activities of saliva on the host inflammation and immunity remains limited.

Cell-based assays have been used to elucidate the immunomodulatory effects of *Ae. aegypti* saliva or salivary gland extract (SGE) preparations *in vitro*. For example, the incubation of rat mast cells with *Ae. aegypti* SGE decreased the release of TNF-α in response to several stimuli whereas no changes were observed for histamine release (14). The proliferation and the production of pro-inflammatory and Th1 cytokines by activated murine T lymphocytes were inhibited in the presence of *Ae. aegypti* SGE (15–17). This inhibition seems to be a consequence of T lymphocyte death that occurs in a caspase-3- and caspase-8-dependent manner (18). For dendritic cells, most studies failed to show direct effects of *Ae. aegypti* saliva/SGE on these cells, irrespective of the source (murine or human) and phenotype evaluated (17–20). On the other hand, *Ae. aegypti* SGE downmodulated the expression of inflammatory mediators and microbicidal functions in murine macrophages under different stimuli. Decreased levels of mRNA to interferon-β (IFN-β) and inducible nitric oxide synthase (iNOS) were expressed by macrophages infected with West Nile virus or Sindbis virus in the presence of *Ae. aegypti* SGE (20). Under M1 polarization (LPS plus IFN-γ stimulation), murine macrophages pre-incubated with *Ae. aegypti* SGE produced less nitric oxide, IL-6 and IL-12 and more IL-10. This phenotype was associated with inhibition of iNOS and phosphorylated nuclear factor-κB (NF-κB) (21). Likewise, murine macrophages activated with LPS and incubated with cecropins (antimicrobial peptides identified in the *Ae. aegypti* genome) presented a decreased production of nitric oxide and inflammatory cytokines, as well as inhibited expression of iNOS, mitogen-activated protein kinases and NF-κB (22). Together, these complex and selective activities may be related to the ability of *Ae. aegypti* saliva and its molecules to modulate cells of the immune system and, therefore, facilitate the transmission of certain types of arboviral infections (23–28).

The transcriptomes of salivary glands (sialotranscriptomes) have revolutionized the study of the proteins present in *Ae. aegypti* saliva (29–31). The evolution of the sequencing platforms allowed the identification of over 200 transcripts that are overexpressed in female salivary glands in comparison to male salivary glands, suggesting that these products are involved in blood feeding and, consequently, with modulation of hemostatic and/or immune functions of the vertebrate host. Among the transcripts identified in this category, a member of the 9.7 kDa polypeptide family presented 30-fold increased expression in female salivary glands when compared with male salivary glands (31). The sequence was annotated as ‘7.8 kDa secreted protein’ and its protein product has been mapped in female *Ae. aegypti* salivary gland lobes (32). In the present work, we confirmed the presence of the peptide in *Ae. aegypti* saliva, determined its selective immunomodulatory activities on macrophages and showed its potential to treat experimental inflammatory bowel disease. Based on all these features, we named it ***Ae*** *des*-specific **MO**dulatory **PE**ptide (AeMOPE-1) and propose its potential use in clinical applications for the treatment of autoimmune disorders.

## Material and Methods

### Animals

Female C57BL/6 mice (6-8 weeks old) were bred and maintained in the Isogenic Breeding Unit, at Institute of Biomedical Sciences, University of Sao Paulo (ICB/USP). Male C57BL/6 mice (6-8 weeks old) were bred and maintained in the animal facility at School of Pharmaceutical Sciences of Ribeirao Preto, University of Sao Paulo (FCFRP/USP). All experiments involving laboratory animals were performed under specific pathogen-free conditions and evaluated by the Institutional Animal Care and Use Committees and approved under the protocol number 140/2011 (ICB/USP) and 11.1.1080.53.1 (FCFRP/USP). The procedures are according to the Brazilian National Law number 11794, which regulates the research activities involving animal use.

### *Aedes aegypti* 7.8 kDa Peptide (AeMOPE-1)

The mature sequence of AeMOPE-1 was synthesized by Dr. Jan Lukszo from Peptide Synthesis and Analysis Laboratory, Research Technologies Branch, National Institute of Allergy and Infectious Diseases, National Institutes of Health (NIAID/NIH) and used in most experimental assays. The whole sequence is found at NCBI GenBank, under the accession AF466588.1 and the sequence follows: MSYWRNNYIIFIAVIIVGSQLTAWAESDVEKYCKYLDCKGGRVKMGESFAATKFAFGYCTCGEENGKKYTRYLPCNFGDTFSLEQQKCVKGVAKA (signal peptide is underlined). The synthetic AeMOPE-1 (syn-AeMOPE-1) batches were suspended in pure dimethyl sulfoxide (DMSO - Sigma-Aldrich, St. Louis, MO, USA) at a 2 mM concentration, aliquoted and stored at -80° C until use. Care was taken to avoid oxidation of the methionine, leading to a low yield of the synthesis. Work solutions were prepared by diluting the concentrated material in appropriated culture medium or buffer (indicated for each case). In all assays performed, DMSO was added to the culture medium or buffer as a negative control at the same dilution used for the highest concentration of syn-AeMOPE-1 (1:1000).

### Salivary Gland Extract

*Aedes aegypti* mosquitoes were bred and maintained in an insectary at the Department of Parasitology, ICB/USP, Brazil as previously described (33). Salivary glands from female mosquitoes 5-7 days old were dissected in phosphate-buffered saline (PBS) and transferred to a microtube containing 50 μL of cold PBS. After that, salivary glands were sonicated to release the soluble material and centrifuged at 14,000 *g* for 10 minutes to remove particulate material. The supernatant was sterilized by passing through a nitrocellulose membrane filter with 0.22 µM pores (Millipore, Billerica, MA, USA) and the protein concentration was measured by NanoDrop 2000 (Thermo Fisher Scientific, Wilmington, DE, USA). Finally, SGE aliquots were stored at -80°C until use.

### Immunoreactivity of AeMOPE-1

Mice were immunized with syn-AeMOPE-1 (20 µg/animal) emulsified in adjuvant as described (34). Two weeks after the last immunization, mice received an i.v. booster of syn-AeMOPE-1 (0.2 μg in 100 μL). Serum was separated from blood collected of mice before the immunization (pre-immune serum) and 3 days after the syn-AeMOPE-1 booster (immune serum). For evaluation of immunoreactivity, syn-AeMOPE-1 (0.1, 0.5 and 1 μg) and *Ae. aegypti* SGE (10 and 40 μg) were mixed with Bolt™ Sample Reducing Agent and Bolt™ LDS Sample Buffer (Invitrogen, Carlsbad, CA, USA) and heated to 70°C for 10 minutes. Proteins were separated on Bolt™Bis-Tris Plus Gels 4-12% and transferred to a nitrocellulose membrane using iBlot® Dry Blotting System (Invitrogen). Membranes were blocked for 2 hours with 10% fetal bovine serum diluted into Tris Buffer, pH 7.5 containing 0.1% Tween-20 (TBST), washed with TBST, and incubated overnight at 4°C with the non-immune and immune sera (1:100 dilution). After further washing, the membranes were incubated 1 hour at room temperature with anti-mouse IgG secondary antibodies (1:3,000) conjugated with horseradish peroxidase for detection (Cell Signaling Technology Inc., Danvers, MA, USA). Immunoreactive bands were stained using the Novex^®^ Chemiluminescent Substrate Reagent Kit (Invitrogen) and visualized in a G:BOX photodocumentation system (Syngene, Cambridge, UK).

Mice were also sensitized to mosquito bites through the methodology described by Barros et al. (13) with modifications. Briefly, each mouse was anesthetized and maintained for 30 minutes on the top of individual plastic containers covered with tulle fabric and containing 50 female mosquitoes. This procedure was performed ten times with a 14-day interval between the exposures and the proportion of mosquitoes fed at each sensitization was typically 80%. One day before each exposure, blood samples were collected and sera were separated and stored at -80°C. For evaluation of immunoreactivity, ELISA plates were coated with syn-AeMOPE-1 (0.5 µg/well), incubated with serum from animals exposed 0 to 10 times to female mosquito bites (1:100 dilution) and then with secondary antibody anti-mouse IgG conjugated with HRP. The plates were revealed after substrate addition and read in a spectrophotometer at a 450 nm wavelength.

### Fractionation of SGE Proteins

A SGE preparation (1 mg) was fractionated by high performance liquid chromatography (HPLC) using column Superdex Peptide HR 10/30 (Pharmacia Biotech, Uppsala, Sweden) linked to a chromatography AKTA Purifier HPLC System (Amersham Biosciences, Piscataway, NJ, USA). This column provides chromatographic resolution for peptides. Chromatography was performed using PBS for 60 minutes in a flow rate of 0.5 mL/minute and the absorbance were monitored at 225 and 280 nm wavelengths. Fractions were collected, aliquoted and stored at -80°C until use.

### T Lymphocyte Proliferation

Spleens were aseptically removed and a cell suspension containing 10^6^ cells/mL was prepared in complete medium (RPMI 1640; 2 mM L-glutamine; 25 mM Hepes; 10% fetal bovine serum; 2.5 × 10^-5^M 2-mercaptoethanol; 100 U/mL penicillin; 100 μg/mL streptomycin – all from Gibco-Invitrogen, Grand Island, NY, USA). Cells were added to 96-wells plates in aliquots of 100 µL/well and pre-incubated overnight with syn-AeMOPE-1 (0.25 to 2 µM). After that, concanavalin A (Sigma-Aldrich) – a polyclonal activator of T lymphocytes – was used to stimulate the cells at 0.5 µg/mL for 72 hours at 37°C and 5% CO_2_. Proliferation of T lymphocytes was evaluated by adding 0.01% resazurin (Sigma-Aldrich) in the last 24 hours incubation, as previously described (18, 34).

### Differentiation and Maturation of Bone Marrow-Derived Dendritic Cells

Bone marrow cells were collected from femurs of mice and cultured at 3 × 10^5^ cells/mL in complete medium and 20 ng/mL of murine GM-CSF. After 4 days of incubation, half the volume was removed and replaced by an equal volume of complete medium containing GM-CSF. At 6 days of incubation, non-adherent cells were collected, suspended at 10^6^ cells/mL, and distributed into 24-well plates in aliquots of 1 mL/well. These cells were pre-incubated overnight with syn-AeMOPE-1 (0.25 to 2 µM), followed by stimulation with 100 ng/mL ultrapure LPS from *Escherichia coli* 0111:B4 strain (InvivoGen, San Diego, CA, USA) – a highly specific TLR4 ligand – for 24 hours to promote their maturation. Cells were transferred into 12 × 75 mm polypropylene tubes and stained with fluorochrome-conjugated antibodies anti-CD11c, anti-MHC class II, anti-CD80 and anti-CD86 (BD Biosciences, San Jose, CA, USA). Cells were acquired in a FACSCanto II (BD Biosciences) and analysis were performed using FlowJo software, version 10.0.5 (Tree Star, Ashland, OR, USA). The results were expressed as the percentage of positive cells for each marker.

### Macrophage Activation

Peritoneal macrophages were elicited by intraperitoneal injection of 3% thioglycollate (BD-Difco, Franklin Lakes, NJ, USA) and after 4 days the cells were harvested by peritoneal lavage with cold PBS. A cell suspension containing 2 × 10^6^/mL was prepared and then aliquots of 100 µL/well were added to 96-well plates and incubated for 2 hours at 37°C and 5% CO_2_. Next, non-adherent cells were removed by washing the plates 3 times with warm PBS, and the adherent cells were considered macrophages. These cells were pre-incubated overnight with DMSO (1:1000 dilution) or syn-AeMOPE-1 (0.25 to 2 µM) and stimulated with ultrapure LPS (10 ng/mL – InvivoGen) and recombinant murine IFN-γ (10 ng/mL - Sigma-Aldrich). In another set of experiments, macrophages were pre-incubated overnight with different DMSO dilutions (1:1000, 1:500, 1:200, 1:100, 1:50, 1:20) and stimulated with ultrapure LPS and IFN-γ. After 48 hours, the supernatants were collected and stored at -80°C until use.

Macrophages were pre-incubated with DMSO (1:1000 dilution) or syn-AeMOPE-1 (2 µM) and stimulated with ultrapure LPS and IFN-γ for 24 hours. Then, cells were transferred into 12 × 75 mm polypropylene tubes and stained with stained with anti-F4/80, anti-CD40, anti-CD80 and anti-CD86 fluorochrome-conjugated antibodies. Cells were acquired in a FACSCalibur (BD Biosciences) and analysis were performed using FlowJo software as described above. The results were expressed in percentage of positive cells for each marker.

### Nitric Oxide Quantification

Nitric oxide production was detected in macrophages supernatants through Griess reaction as previously described (35). Macrophage supernatants (50 µL) were mixed with the same volume of Griess reagent (solution A: 1% sulfanilamide in 5% H_3_PO_4_ and solution B: 0.1% *N*-(1-Napthyl) ethylenediamine dihydrochloride). Absorbance was measured at 554 nm wavelength and the results were calculated from a standard curve (NaNO_2_) and expressed in µM/2 × 10^5^ cells.

Nitric oxide levels were also evaluated in gut segments collected at the endpoint of the colitis assay. The segments were weighted, homogenized in 500 µL of PBS, and centrifuged at 10,000 *g* for 15 minutes at 4°C. The supernatants (50 µL) were mixed with the same volume of Griess reagent and absorbance was measured as described above. The results were calculated from a standard curve (NaNO_2_) and expressed in µM/mg of protein.

### Macrophage Viability

Macrophages were prepared as described above and incubated with DMSO (1:1000 dilution) or AeMOPE-1 (2 µM) for 4 hours. Cells were transferred into 12 x 75 mm polypropylene tubes and stained with anti-F4/80 fluorochrome-conjugated antibodies. Cell viability was evaluated by incubation with Live/Dead Viability/Cytotoxicity Assay Kit (ThermoFisher Scientific), followed by acquisition in a FACSCanto II and analysis using FlowJo software as described above. The results were expressed as the percentage of positive cells for each marker.

Macrophages were incubated with DMSO (1:1000 dilution) or AeMOPE-1 (2 µM) in the presence of 0.01% resazurin (Sigma-Aldrich) prepared in complete medium and added to the culture at 10% of the total volume. Cell viability was evaluated after 3, 6, 15 and 24 hours by reading the absorbance of the culture plates at 570 and 600 nm wavelengths. The results are expressed as the difference between those readings as described (18, 34).

### Evaluation of Intracellular Proteins

Macrophages were prepared as describe above and lysed with RIPA buffer (150 mM NaCL, 1% NP40, 0.1% SDS, 50 mM Tris, pH 8.0) supplemented with phosphatase inhibitors (100 mM sodium fluoride and 100 mM sodium orthovanadate) and protease inhibitor 1% (Sigma-Aldrich). Cell lysate was centrifuged at 16,000 *g* for 10 minutes and the protein concentration was measured by BCA Protein Assay Kit (Thermo Fisher Scientific), according to the manufacturer’s instructions. Next, volumes of each sample containing the same amount of protein were mixed with Bolt™ Sample Reducing Agent and Bolt™ LDS Sample Buffer and heated to 70°C for 10 minutes. Gel electrophoresis separation, transfer to nitrocellulose membranes and membrane blockage were performed as described earlier. After washing with TBST, the membranes were incubated overnight at 4°C with the following monoclonal antibodies: anti-iNOS (1:10,000), anti-IκBα (1:1,000), anti-phospho-NF-κB p65 (1:1,000) and anti-phospho p38 MAP Kinase (1:500) (Cell Signaling Technology Inc.) After further washing, the membranes were incubated 1 hour at room temperature with anti-rabbit secondary antibodies (1:1,000) conjugated with horseradish peroxidase for detection. Immunoreactive bands were stained and visualized in a photodocumentation system as described earlier. The membranes were then washed and incubated 1 hour with anti-β-actin conjugated with horseradish peroxidase (1:10,000) (Dako, Glostrup, Denmark) and immunoreactive bands were also stained and visualized in a photodocumentation system. The density of the bands was analyzed using AlphaDigiDoc™ System 1000 software version 3.2 Beta (Alpha Innotech Corp., San Leandro, CA, USA). The values were normalized by the total β-actin present in each sample and expressed in percentage in relation to negative control.

### Colitis Induction, Treatment and Scoring

Male C57BL/6 mice were divided into groups of five animals each. For colitis development, animals received 3% of dextran sulfate sodium (DSS - MP Biomedicals, Illkirch, France) uninterruptedly in their drinking water for 6 consecutive days. For colitis treatment, animals received syn-AeMOPE-1 (1 µg/animal i.v.) or DMSO (as a control), both diluted in 0.9% saline, from 3^rd^ until 5^th^ days and were euthanized at day 6 after the beginning of DSS exposure. The treatment was initiated only after the appearance of the first signs of intestinal inflammation, evaluated daily by clinical parameters used for calculating the disease clinical scores. The signs observed were as follow: weight loss; wet anus; diarrhea; bleeding stools; hypoactivity and piloerection. It was attributed one point to each sign presented by the animal. The animals that lost 5 to 10% of body weight from one day to another received one point, the animals that lost more than 10% of body weight received two points. The sum of daily scores was computed and was represented as a daily clinical disease score. To better represent the animal clinical condition during the entire period of disease induction, the sum of all daily clinical scores of each animal was computed thus representing an overall clinical score. After disease induction the animals were euthanized and the macroscopic aspects of colon were evaluated. The parameters evaluated were: presence of diarrhea and bleeding, local or diffuse and stenosis. To each sign were attributed one point to local and two to diffuse. The final score was obtained by the sum of signs presented by each animal and was represented as post-mortem score (36). The intestines (colon) were collected and frozen immediately in TRIzol^®^ Reagent (Life Technologies, Carlsbad, CA, USA) for quantification of cytokine expression. Blood samples were collected and smears were stained with Romanowsky stain (Laborclin, Pinhais, PR, Brazil) to differential count of blood cells and total leukocytes were counted using a Neubauer chamber.

### RT-qPCR

*Aedes aegypti* life stages were collected as follow: larvae (L1 to L4), male and female pupae, male and female adult mosquito thorax, abdomen, and salivary glands. The total RNA was extracted using the TRIzol^®^ Reagent and 1 µg was used as template for the reverse transcription in cDNA using Platus transcriber RNase H cDNA First Strand Kit (Sinapse – Inc, Miami, FL, USA), according with the manufacturer. Resulting cDNA was subjected to qPCR using sense and antisense oligonucleotides designed with base in the DNA sequence previously reported (30) or the S7 ribosomal protein. The reactions were performed as previously described (37) and the expression of AeMOPE-1 gene in different tissues of mosquito was calculated using the 2^-ΔΔCT^ method, as proposed previously (38) and considering the larvae L1 as reference.

Gut samples were collected at the endpoint of the colitis assay. The total RNA was extracted using the TRIzol^®^ Reagent and 1 µg was used as template for the reverse transcription in cDNA using the High Capacity kit (Applied Biosystems, Foster City, CA, USA) following the manufacturer’s instructions. The reactions were performed as previously described (39) and the expression of IL-6, IFN-γ and CCL2 genes in gut samples was calculated using the 2^-ΔΔCT^ method and compared with the expression of the housekeeping gene β-actin.

### Cytokine Evaluation

The cytokines IL-6, IL-12p40, CCL2 and TNF-α were detected in supernatants of macrophage cultures. The levels of cytokines were quantified by ELISA (OptEIA™ Set – BD Biosciences) according to the manufacturer’s instructions.

### Quantification of Macrophage Activity

To indirectly evaluate macrophage infiltration and function in the colon of mice treated with syn-AeMOPE-1, the local levels of *N*-acetyl-β-D-glucosaminidase (NAG), a marker of macrophage activity, was evaluated according to Bento et al. (40), with some modifications. Briefly, gut segments collected at the endpoint of the colitis assay were weighted, homogenized in 500 µL of PBS, and centrifuged at 10,000 *g* for 15 minutes at 4°C. The NAG assay was performed in 96-well plates by incubating 25 µL of the supernatant (1:10 dilution) with 25 µL of 2.25 mM of *p*-nitrophenyl-2-acetamide β-D-glucopyranoside for 1h at 37°C, followed by 100 µL of 50 mM citrate buffer (pH 4.5) to block the reaction. The absorbance was read at 405 nm wavelength for determining NAG activity and the results were expressed as optical density relative to tissue weight (O.D./mg).

### Statistical Analyses

Statistical analyses of differences between means of experimental groups was performed using Student’s *t* test (for two groups comparisons) or analysis of variance (ANOVA) followed by Tukey as a post-test (for three or more groups). A value of *P *≤ 0.05 was considered statistically significant.

## Results

### Peptide Sequence, Presence in Saliva and Antigenicity

**Figure 1:**
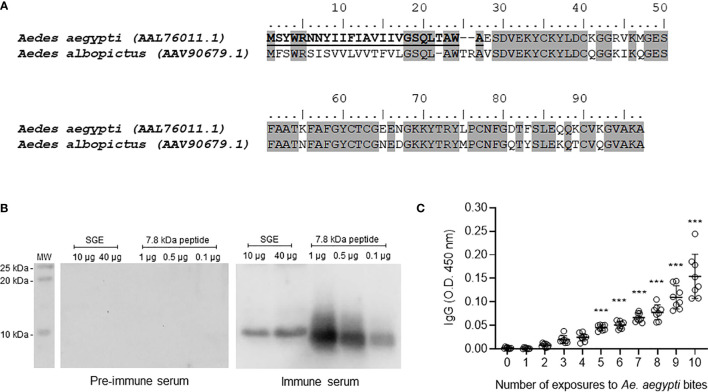
*Ae. aegypti* 7.8 kDa peptide is secreted in the mosquito saliva and triggers the production of specific antibodies following exposure to mosquitoes. **(A)** Amino acid sequence and alignment of *Ae. aegypti* (Accession number AAL76011.1) and *Ae. albopictus* (Accession number AAV90679.1) 7.8 kDa salivary peptides. The alignment was performed using MUSCLE method and graphically edited using BioEdit software. Gray highlights the identical amino acid residues (68% identity) and bold underlined amino acid residues indicate the signal peptide. **(B)** *Ae. aegypti* salivary gland extract (SGE - 10 and 40 µg) and 7.8 kDa salivary peptide (0.1, 0.5 and 1 µg) were submitted to electrophoresis on a 15% polyacrylamide gel under reducing conditions. Western blot was performed according to Material and Methods to test the reactivity of pre-immune and immune serum. The immunoreactive bands were revealed with luminescence substrate and acquired by an imaging system. **(C)** Sera of mice repeatedly exposed to female *Ae. aegypti* bites recognize the salivary peptide. Plates were coated with the salivary peptide (0.5 µg/well), incubated with serum from animals exposed 0 to 10 times to female mosquito bites (1:100 dilution) and then with secondary antibody anti-mouse IgG conjugated with HRP. The plates were revealed after substrate addition and read in a spectrophotometer at a 450 nm wavelength. White circles in **(C)** indicate individual sera for each exposure (n = 8). The mean ± SEM are indicated. ***P ≤ 0.001 *versus* group “0” (not exposed to mosquito bites) – One-way ANOVA.

The sialotranscriptome of *Ae. aegypti* revealed an overexpressed sequence in female salivary glands coding for a member of the 9.7 kDa family, originally classified as ‘other *Aedes* specific polypeptides’, whose function was unknown (29). Its polyadenylated cDNA (NCBI accession AF466588.1) has 739 nucleotides coding for a small protein of 95 amino acids, including a signal peptide of 25 amino acids, a predicted mature protein with molecular weight of 7.84 kDa and a basic isoelectric point of 8.56. No *N*-glycosylation or *O*-galactosylation sites were predicted for this peptide according to the NetNGlic 1.0 and NetOClyc 4.0 Servers (41). No tyrosine sulfation was predicted in the peptide sequence according to Sulfinator (https://web.expasy.org/sulfinator/). BLASTp searches revealed 68% identity with a putative 7.8 kDa secreted peptide from *Aedes albopictus* (**Figure 1A**). Because no other sequence homology was close enough for the modelling programs to work with, the attempts to produce a reliable three-dimensional structure of the peptide were unsuccessful. A number of these models suggest that four of the six cysteines are in position to form disulfide bonds. However, none of the models achieved enough score to be displayed (data not shown).

The peptide gene expression was detected in tissues of adult mosquitoes, but not pupae or larval stages. In addition, the gene expression was much higher in female tissues compared with male tissues. As expected, thorax (where the salivary glands are located) and salivary glands of female mosquitoes presented high gene expression of 7.8 kDa peptide and no expression was detected in abdomen samples (**Supplementary Figure 1**).

The mature secreted form of the peptide was synthetized and used to immunize mice for antibody generation. Pre-immune and immune sera were evaluated by Western Blot to detect reactive bands in salivary preparations. **Figure 1B** shows that immune serum recognized a single band nearly the 10 kDa molecular weight marker in both SGE and peptide lanes. As expected, pre-immune serum did not recognize either the naturally secreted or the syn-AeMOPE-1. Next, we demonstrated that the peptide naturally secreted in *Ae. aegypti* saliva was able to trigger an increasing production of specific antibodies after repeated exposure to the mosquitoes which was significant after the 5^th^ exposure (**Figure 1C**). These results confirm the presence and immunogenicity of the peptide in *Ae. aegypti* saliva.

### *Aedes aegypti* Salivary Peptide Modulates Macrophage, But Not Lymphocyte or Dendritic Cell Biology

**Figure 2:**
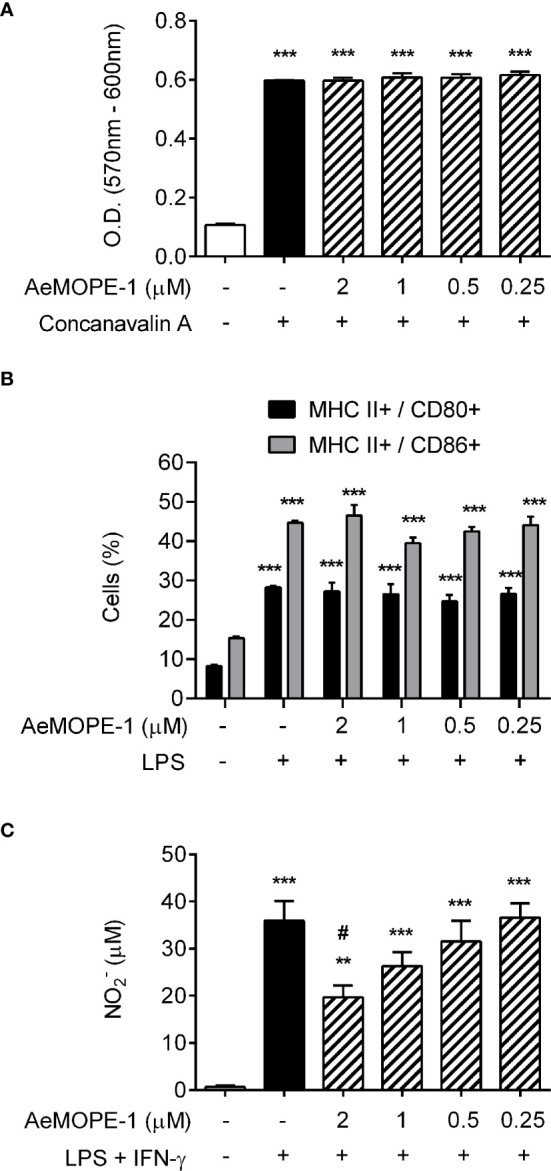
Syn-AeMOPE-1 decreases macrophage production of nitric oxide but does not affect proliferation of T lymphocytes or dendritic cell maturation. **(A)** Total spleen cells were pre-incubated with syn-AeMOPE-1 (0.25 - 2 µM), stimulated with concanavalin A (0.5 µg/ml) for 72 hours, and the proliferation of T lymphocytes was measured by a colorimetric assay. Results are expressed as the mean ± SEM from one representative experiment of three independent experiments assayed in sextuplicate. **(B)** Bone marrow-derived dendritic cells were pre-incubated with syn-AeMOPE-1 (0.25 - 2 µM), stimulated with LPS (100 ng/ml) for 24 hours, and the percentage of MHC class II^+^ CD80^+^ or MHC class II^+^ CD86^+^ cells were evaluated by flow cytometry. Gating strategy and representative plots are presented in **Supplementary Figure 2**. Results are expressed as the mean ± SEM from one representative experiment of four independent experiments assayed in quadruplicate. **(C)** Thioglycolate-elicited peritoneal macrophages were pre-incubated with syn-AeMOPE-1 (0.25 - 2 µM), stimulated with LPS+IFN-γ (10 ng/mL of each) for 48 hours, and nitric oxide production was measured by Griess reaction. Results are expressed as the mean ± SEM from one representative experiment of five independent experiments assayed in duplicate. **P ≤ 0.01 and ***P ≤ 0.001 *versus* unstimulated/untreated cells; ^#^ *P *≤ 0.05 *versus* LPS+IFN-γ-stimulated cells – One-way ANOVA.

In an attempt to identify putative functions of the salivary peptide, we have evaluated its effect in different cell-based assays. Under our experimental conditions, the peptide did not interfere with mitogen-induced T lymphocyte proliferation (**Figure 2A**) or LPS-induced dendritic cell maturation (**Figure 2B** and **Supplementary Figure 2**) at any of the concentrations employed. On the other hand, the production of nitric oxide by macrophages was inhibited in a concentration-dependent manner when the cells were preincubated with the peptide and then stimulated with IFN-γ plus LPS. Nonetheless, this inhibition was significant only in the presence of the highest concentration (2 µM) of the peptide (**Figure 2C**). Given the biological activity of the 7.8 kDa salivary peptide, we named it ***Ae*** *des*-specific **MO**dulatory **PE**ptide 1 (AeMOPE-1).

**Figure 3:**
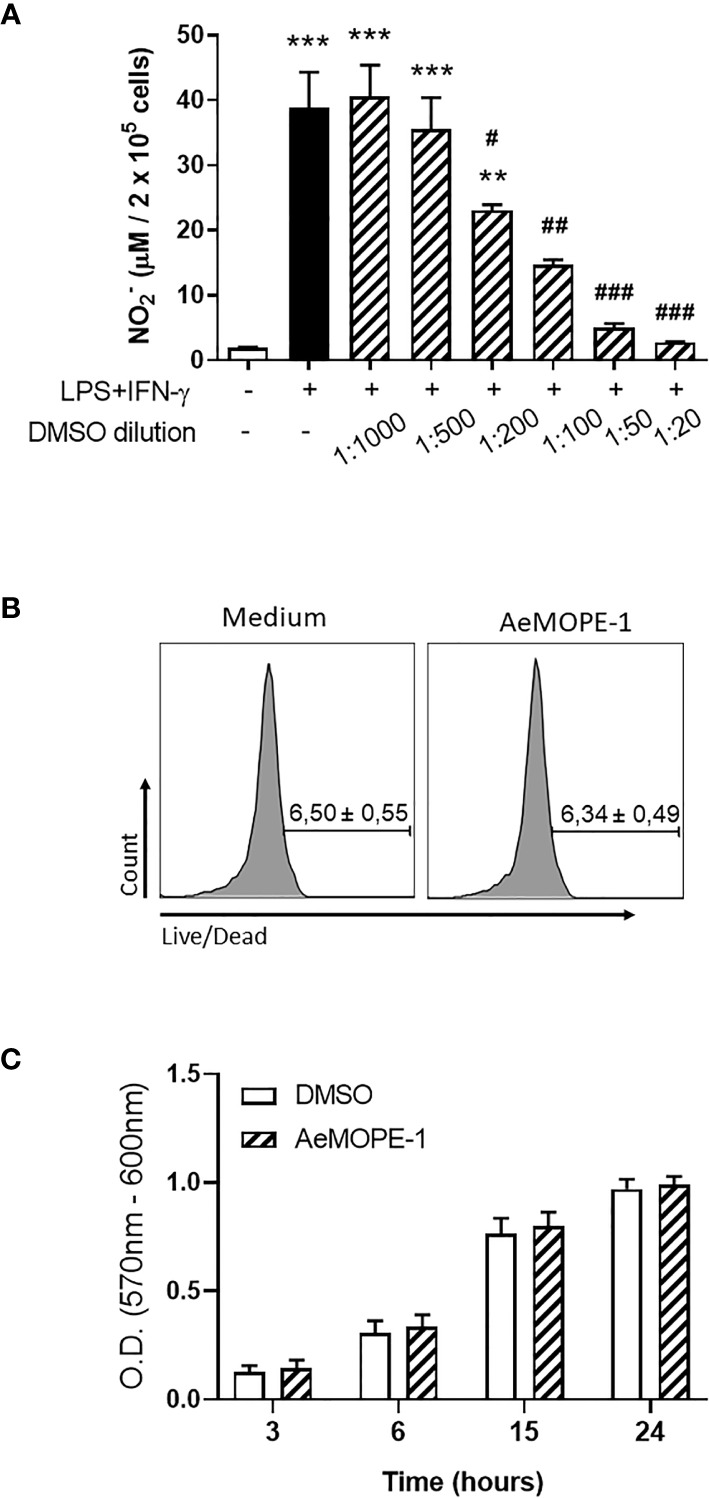
Neither DMSO nor syn-AeMOPE-1 are cytotoxic to macrophages at the dilution used in the experiments. **(A)** Thioglycolate-elicited peritoneal macrophages were pre-incubated with increasing concentrations of DMSO (indicated in the figure), stimulated with LPS+IFN-γ (10 ng/mL of each) for 48 hours, and nitric oxide production was measured by Griess reaction. **(B)** Thioglycolate-elicited peritoneal macrophages were incubated with DMSO (1:1000) or AeMOPE-1 (2 µm) for 4 hours, followed by staining with Live/Dead Viability/Cytotoxicity Assay Kit and evaluation by flow cytometry. Gating strategy and representative plots are presented in **Supplementary Figure 3A**. **(C)** Thioglycolate-elicited peritoneal macrophages were incubated with DMSO (1:1000) or AeMOPE-1 (2 µm) for 24 hours and the basal metabolic activity was monitored at different time points by a colorimetric assay. Results are expressed as the mean ± SEM from three **(A, B)** or five **(C)** independent experiments assayed in duplicates **(A, B)** or triplicates **(C)**. **P ≤ 0.01 and ***P ≤ 0.001 *versus* unstimulated/untreated cells; ^#^ *P *≤ 0.05, ^##^ *P *≤ 0.01 and ^###^ *P *≤ 0.001 *versus* LPS+IFN-γ-stimulated cells – One-way ANOVA.

Because syn-AeMOPE-1 was solubilized in DMSO and the effective concentration represented a 1:1000 dilution from the stock solution, macrophages were preincubated with increasing amounts of DMSO or medium only (as a control), followed by stimulation with LPS+IFN-γ. In the presence of higher DMSO dilutions (1:1000 and 1:500), no significant differences were observed in nitric oxide production compared to the control (**Figure 3A**). On the other hand, lower dilutions of DMSO (1:200, 1:100, 1:50 and 1:20) seemed toxic to the cells since a concentration-dependent inhibition of nitric oxide was observed in the culture supernatants (**Figure 3A**). Accordingly, the viability of macrophages was not changed upon incubation with 2 µM of syn-AeMOPE-1 as measured by flow cytometry (**Figure 3B**) or by a colorimetric assay (**Figure 3C**). Together, this data supports the notion that syn-AeMOPE-1 is immunomodulatory rather than cytotoxic to macrophages.

**Figure 4:**
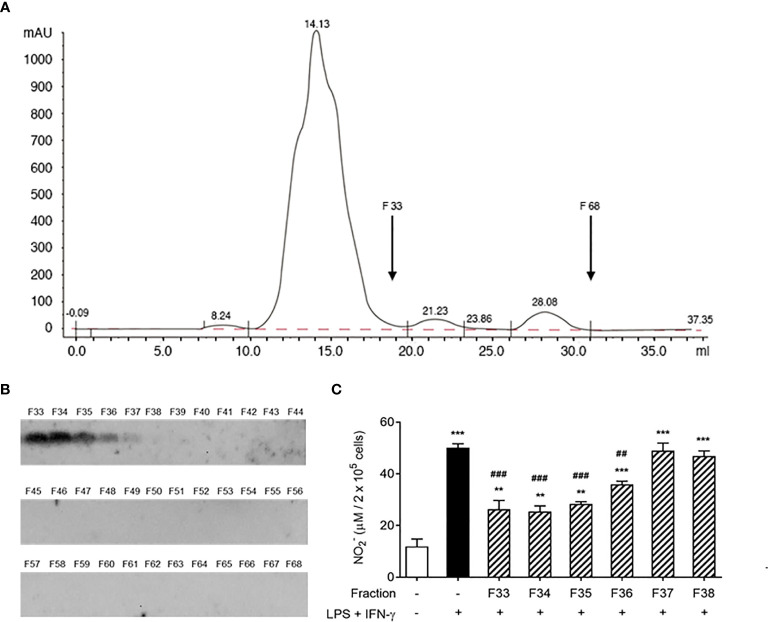
Native salivary AeMOPE-1 inhibits nitric oxide production by macrophages. **(A)** SGE was fractionated by HPLC using a column that resolves peptides, fractions were eluted at 0.5 ml/minute and their absorbance monitored at 280 nm wavelength. The arrow indicates the range of low molecular fractions (F33 to F68). **(B)** Western Blot of F33 to 68 revealed with serum from animals immunized with syn-AeMOPE-1. **(C)** Macrophages were pre-incubated with each fraction from F33 to F38 (representing the low molecular weight fractions), stimulated with LPS + IFN-γ (10 ng/mL of each) for 48 hours, and nitric oxide production was measured by Griess reaction. Results are expressed as the mean ± SEM from three independent experiments assayed in duplicate. **P ≤ 0.01 and ***P ≤ 0.001 *versus* unstimulated/untreated cells; ^##^ *P *≤ 0.01 and ^###^ *P *≤ 0.001 *versus* LPS+IFN-γ-stimulated cells – One-way ANOVA.

Next, we tested whether the AeMOPE-1 naturally found in mosquito saliva would present the same biological function found for the synthetic peptide. For this, SGE was fractionated by HPLC with a column used to resolve peptides (Superdex Peptide HR 10/30). The chromatogram presented in **Figure 4A** shows that the fractionation successfully separated larger molecules (tall peak eluted from 11 to 18 mL) from small molecules (short peaks eluted from 19 to 31 mL). Thus, the low molecular weight fractions (F33 to F68) were individually blotted against the serum of mice immunized with syn-AeMOPE-1 (**Figure 4B**). The native peptide was strongly recognized in the fractions F33 to F36, which were then tested in the nitric oxide production assay. **Figure 4C** reveals that these positive fractions were able to negatively modulate nitric oxide production by macrophages.

### Syn-AeMOPE-1 Affects Parameters of Macrophage Activation in a Selective Manner

**Figure 5:**
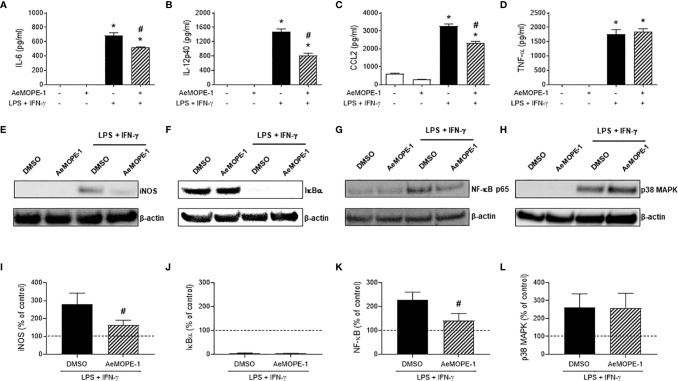
Syn-AeMOPE-1 selectively modulates macrophage activation. Macrophages were pre-incubated with syn-AeMOPE-1 (2 µM) and stimulated with LPS + IFN-γ (10 ng/mL of each). After 48 hours the production of cytokines **(A)** IL-6, **(B)** IL-12p40, **(C)** CCL2, and **(D)** TNF-α were detected in supernatants by ELISA. Values are expressed as the mean ± SEM (n = 4). *P ≤ 0.0001 *versus* unstimulated/untreated cells; ^#^ *P *≤ 0.0001 *versus* LPS+IFN-γ-stimulated cells – One-way ANOVA. Representative Western blots and densitometry for **(E, I)** iNOS, **(F, J)** IκBα, **(G, K)** NF-κBp65, and **(H, L)** p38-MAPK performed with cell lysates at different time points. Results are expressed as the mean ± SEM from three independent experiments. ^#^ *P *≤ 0.05 *versus* LPS+IFN-γ-stimulated cells –Student’s *t* test.

The role of syn-AeMOPE-1 on cytokine production, cell surface markers and intracellular protein expression by activated macrophages was explored. We observed that the presence of syn-AeMOPE-1 in resting macrophage cultures did not affect the basal levels of the above-mentioned parameters. On the other hand, macrophages activated with LPS plus IFN-γ in the presence of syn-AeMOPE-1 produced significant less IL-6 (**Figure 5A**), IL-12p40 (**Figure 5B**) and CCL-2 (**Figure 5C**) than macrophages maintained in medium containing DMSO only before activation. The production of TNF-α (**Figure 5D**) by activated macrophages was similar in the presence or absence of the peptide. Moreover, syn-AeMOPE-1 did not affect the expression of the cell surface molecules CD40, CD80 and CD86 either in resting or in activated macrophages (**Supplementary Figure 3**).

Next, we investigated whether the modulatory activities of syn-AeMOPE-1 were due to the inhibition of the expression of intracellular factors associated with macrophage activation. The inducible nitric oxide synthase (iNOS) expression induced by macrophage activation was diminished in the presence of AeMOPE-1 (**Figure 5E**), corroborating with the lower nitric oxide production detected under similar conditions (**Figures 2** and **4**). Regarding the molecules involved in intracellular signaling, the activation of macrophages with LPS plus IFN-γ induced the degradation of IκBα as expected, but no differences were observed in the presence of syn-AeMOPE-1 (**Figure 5F**). On the other hand, activation of macrophages in the presence of syn-AeMOPE-1 decreased NF-κBp65 (**Figure 5G**) but not p38 MAPK (**Figure 5H**) expression when compared with activated macrophages cultured in the absence of the peptide. The densitometry analysis confirmed the above-mentioned changes in macrophages activated with LPS plus IFN-γ (**Figures 5I–L**, respectively). Neither the expression of cyclooxygenase-2 (COX-2) nor the production of its major metabolite, PGE_2_, were significantly changed in our experimental conditions (data not shown).

### Syn-AeMOPE-1 Ameliorates the Clinical Outcome of Experimental Colitis

**Figure 6:**
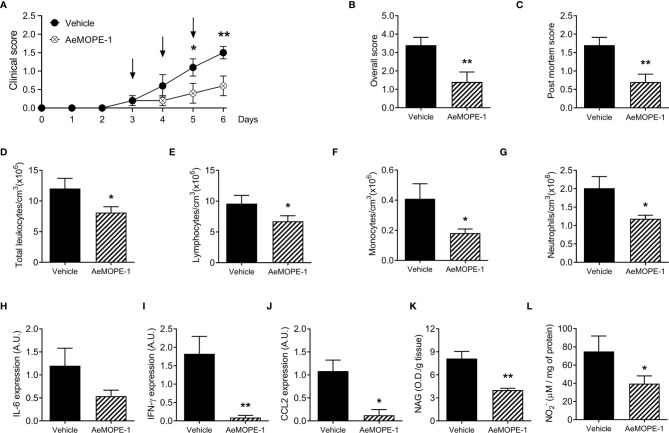
Treatment of mice with syn-AeMOPE-1 ameliorates the clinical signs of experimental colitis. Mice were divided into two groups and received 3% DSS in their drinking water for 6 days. One group was treated daily with syn-AeMOPE-1 (1 µg/animal) while the other received an equal dilution of DMSO, both in 0.9% saline, at the beginning of the clinical signs, as indicated by the arrows. **(A)** Clinical score was assessed daily until the time of euthanasia of animals while **(B)** overall clinical score and **(C)** post-mortem score were evaluated on the day of euthanasia. **(D)** Total circulating leukocytes, **(E)** lymphocytes, **(F)** monocytes and **(G)** neutrophils were counted on the day of euthanasia. Results are expressed as the mean ± SEM (n = 10 animals/group). Data from one representative experiment of two independent experiments are shown. *P ≤ 0.05 and **P ≤ 0.01 *versus* “Vehicle” group –Student’s *t* test. Expression of **(H)** IL-6, **(I)** IFN-γ and **(J)** CCL2 by RT-qPCR, as well as the **(K)** activity of macrophages by NAG quantification and **(L)** nitric oxide levels by Griess reaction, were evaluated in gut homogenates after euthanasia. Results are expressed as the mean ± SEM (n = 4-5 animals/group) from one representative experiment of two independent experiments. *P ≤ 0.05 and **P ≤ 0.01 *versus* “Vehicle” group –Student’s *t* test.

Given the anti-inflammatory effect of syn-AeMOPE-1 on macrophage biology, we next explored the peptide therapeutic potential in an experimental model of inflammatory bowel disease induced by DSS. Mice treated with syn-AeMOPE-1 presented an improvement in the clinical score of the disease when compared to mice that received vehicle only (**Figure 6A**). Likewise, there was a significant reduction in the overall clinical score of the disease (**Figure 6B**). The evaluation of post-mortem parameters showed less diffuse bleeding, diarrhea and better stool consistence in animals treated with syn-AeMOPE-1 when compared with vehicle-treated mice (**Figure 6C**). The total number of circulating leukocytes were decreased in syn-AeMOPE-1-treated mice (**Figure 6D**) and reflected a reduction in lymphocytes (**Figure 6E**), monocytes (**Figure 6F**) and neutrophils (**Figure 6G**) in these animals. The evaluation of cytokine expression in gut homogenates revealed a decrease of IL-6 (**Figure 6H**), IFN-γ (**Figure 6I**) and CCL2 (**Figure 6J**) in the animals treated with syn-AeMOPE-1 when compared with vehicle-treated mice, although the difference did not reach statistical difference in the case of IL-6. Moreover, there was a notable reduction in the macrophage activity in the colon of mice treated with syn-AeMOPE-1 (**Figure 6K**) and nitric oxide levels (**Figure 6L**), indicating that the peptide may influence the accumulation and/or functionality of these cells in the inflamed intestine. Taken together, the results demonstrate that syn-AEMOPE-1 was able to ameliorate clinical signs and immunological parameters of experimental colitis.

## Discussion

The saliva of *Ae. aegypti* and other blood-feeding arthropods is a rich mixture of biologically active components. Although the sequencing and functional characterization of the first *Ae. aegypti* salivary proteins date back to the 1990’s (4, 8, 42), the large-scale elucidation of the mosquito’s sialoverse – the universe of its salivary molecules – is being achieved through the information generated more recently by the species’ sialotranscriptomes (29–31). The present study used the sialotranscriptome database as a starting point to uncover the biological activities of AeMOPE-1, a 7.8 kDa peptide found in *Ae. aegypti* saliva and whose function was elusive thus far.

The unique sequence of AeMOPE-1 was annotated as a member of ‘other *Aedes*-specific polypeptides’ family. In fact, a sequence derived from *Ae. albopictus* sialome (43) was the only partial match found with AeMOPE-1 (68% identity). Unfortunately, the lack of sequence homology with other known proteins precluded the attempts to generate a consistent three-dimensional structure of the peptide *in silico*. The importance of AeMOPE-1 for the hematophagy is suggested by its higher transcriptional levels in the salivary glands of females when compared to males (31), a difference confirmed in the present work. In addition, AeMOPE-1 triggers an increasing production of specific antibodies in *Ae. aegypti*-exposed mice, confirming its secretion in *Ae. aegypti* saliva. Accordingly, the use of AeMOPE-1 as a possible marker of human exposure to *Ae. aegypti* is currently under investigation by our group.

The putative immunomodulatory activities of a syn-AeMOPE-1 preparation was investigated in cell-based assays, considering major phenotypes associated to each cell type. Syn-AeMOPE-1 did not change the polyclonal proliferation of T lymphocytes induced by concanavalin A, despite reports on *Ae. aegypti* SGE inhibiting the activation of T lymphocytes by inducing cell death (15–18). In addition, syn-AeMOPE-1 did not affect LPS-induced dendritic cell maturation and these results are in accordance with previous studies employing *Ae. aegypti* SGE (17–20). In contrast, the presence of syn-AeMOPE-1 in macrophage cultures activated by LPS plus IFN-γ decreased nitric oxide production in a concentration dependent manner. A similar phenotype has been recently described when macrophages were incubated with *Ae. aegypti* SGE (20, 21). Strikingly, the fractionation of the SGE confirmed that the active fractions containing low molecular weight molecules recognized by an immune serum raised against AeMOPE-1 were associated with the nitric oxide inhibition, strongly suggesting a similar biological effect of the naturally secreted peptide. Of importance, a number of controls were performed to rule out a possible cytotoxic activity of the synthetic peptide or due to DMSO presence (used to solubilize the peptide). DMSO was shown to be toxic to nitric oxide production in concentrations equal or higher than 0.5%. This is a 5-fold increase compared to the amount of DMSO present in 2 µM of AeMOPE-1. In addition, the presence of syn-AeMOPE-1 in cultures of resting macrophage did not affect the basal levels of the parameters evaluated. Similarly, macrophage viability was monitored by flow cytometry and by a resazurin-based assay (21, 34, 44), and no evidence was found concerning AeMOPE-1 toxicity under these conditions.

The literature on the activities of *Ae. aegypti* salivary preparations in macrophages is very scarce. Resident peritoneal murine macrophages infected with West Nile virus or Sindbis virus in the presence of *Ae. aegypti* SGE had the expression of IFN-β and iNOS diminished and the expression of IL-10 increased when compared to cells incubated with the virus only (20). *In vivo*, the co-inoculation of dengue virus with SGE in mice in the presence of enhancing antibodies increased the percentage of infected dermal macrophages (26). A recent study showed that IL-6, IL-12 and nitric oxide production by thioglycolate-elicited peritoneal murine macrophages activated with IFN-γ plus LPS was inhibited in the presence of *Ae. aegypti* SGE, while the production of IL-10 was increased and TNF-α has not changed under similar conditions. The presence of SGE during macrophage activation also reduced expression of NF-κB and iNOS, spare respiratory capacity and impaired bacterial internalization and microbicidal activity by these cells (21). These alterations were consistent with a suppression of the proinflammatory program of M1 macrophages, but no evidence of M2 polarization was observed in the presence of *Ae. aegypti* SGE (21). The syn-AeMOPE-1 largely reproduced the anti-inflammatory profile found with SGE by downmodulating parameters associated with macrophage activation. To date, cecropins were the only *Ae. aegypti* salivary molecules presenting anti-inflammatory activities in murine macrophages (22). Nevertheless, cecropins are part of a large family of antimicrobial peptides whose members from other blood-feeding species have already been shown to present immunomodulatory roles in vertebrate macrophages (45, 46). AeMOPE-1, on the other hand, seems to be an orphan molecule specific from the *Aedes* genus. Importantly, only AeMOPE-1 was demonstrated to be present in the mosquito saliva in a pharmacologically active concentration capable of modulating macrophage biology.

Recent studies have been proposing the use of bioactive molecules present in arthropod saliva as a potential source of immunobiologics to treat many conditions (47–49). Such approach revealed many tick salivary molecules effective in preclinical experimental models for studying of cancer (50, 51), multiple sclerosis (52), arthritis (53), asthma (54, 55), allogeneic hematopoietic stem cell transplantation (56), colitis (57), psoriasis and fibrosis (58). From those, Amblyomin-X from *Amblyomma cajennense* is under clinical trial for treatment of advanced solid tumor (https://clinicaltrials.gov/ct2/show/NCT03120130) while Coversin from *Ornithodoros moubata* is under clinical trial for treatment of paroxysmal nocturnal haemoglobinuria (https://clinicaltrials.gov/ct2/show/NCT02591862). Likewise, two salivary molecules identified in a sand fly species were shown to improve arthritis clinical signs (59, 60). For mosquitoes, protective effects of *Ae. aegypti* SGE or salivary molecules were reported in murine models of sepsis (61), endotoxin shock (22), pharmacologically-induced hepatitis (62) and inflammatory bowel disease (36). Therefore, due to its selective anti-inflammatory effect *in vitro*, we investigated the possible role of syn-AeMOPE-1 in DSS-induced colitis. Remarkably, treatment with syn-AeMOPE-1 ameliorated the signs of colitis, reduced the number of circulating leukocytes and the levels of inflammatory cytokines involved in the disease pathology.

A direct comparison between macrophage *in vitro* assays and a systemic condition such as DSS-induced colitis is not straightforward. Nitric oxide, which was inhibited both *in vitro* and *in vivo* by syn-AeMOPE-1, has a dual role in the pathogenesis of inflammatory bowel disease (IBD). It is well known that nitric oxide and its metabolites are present in the intestinal lumen, plasma and urine of patients with IBD, and its overproduction exacerbates gastrointestinal inflammation (63). On the other hand, iNOS-deficient mice are more susceptible to colitis (64, 65). Thus, it seems that a tight regulation of iNOS expression and nitric oxide production is the key to ameliorate gastrointestinal inflammation, as demonstrated by a number of natural and synthetic agents (63). Inhibition of NF-κB by a number of pharmacological agents also had protective effects in murine models of colitis (66–68). However, NF-κB signaling in intestinal epithelial cells is essential for the maintenance of intestinal homeostasis, as demonstrated by development of spontaneous chronic severe colitis in mice with deficiency of modulators of the canonical NF-κB pathway (69). Despite the decreased expression of NF-κB p65 in the presence of syn-AeMOPE-1, this was not accompanied by changes in the cytoplasmatic degradation of its inhibitor, IκBα. Nevertheless, there are evidences that NF-κB-dependent transcription can be modulated after its translocation to the nucleus by proteasome inhibitors (70). This suggests that syn-AeMOPE-1 is somehow acting in uncommon regulatory pathways not yet explored for arthropod salivary molecules. Despite of that, both *in vitro* and *in vivo* treatments with syn-AeMOPE-1 were associated with a reduction in CCL2, a chemokine produced by the NF-κB pathway and classically known to recruit macrophages to inflamed sites (71). In fact, mice with DSS-induced colitis treated with syn-AeMOPE-1 presented a decreased *in vivo* activity/presence of macrophages, which are fundamental to the IBD pathogenesis (72).

**Figure 7:**
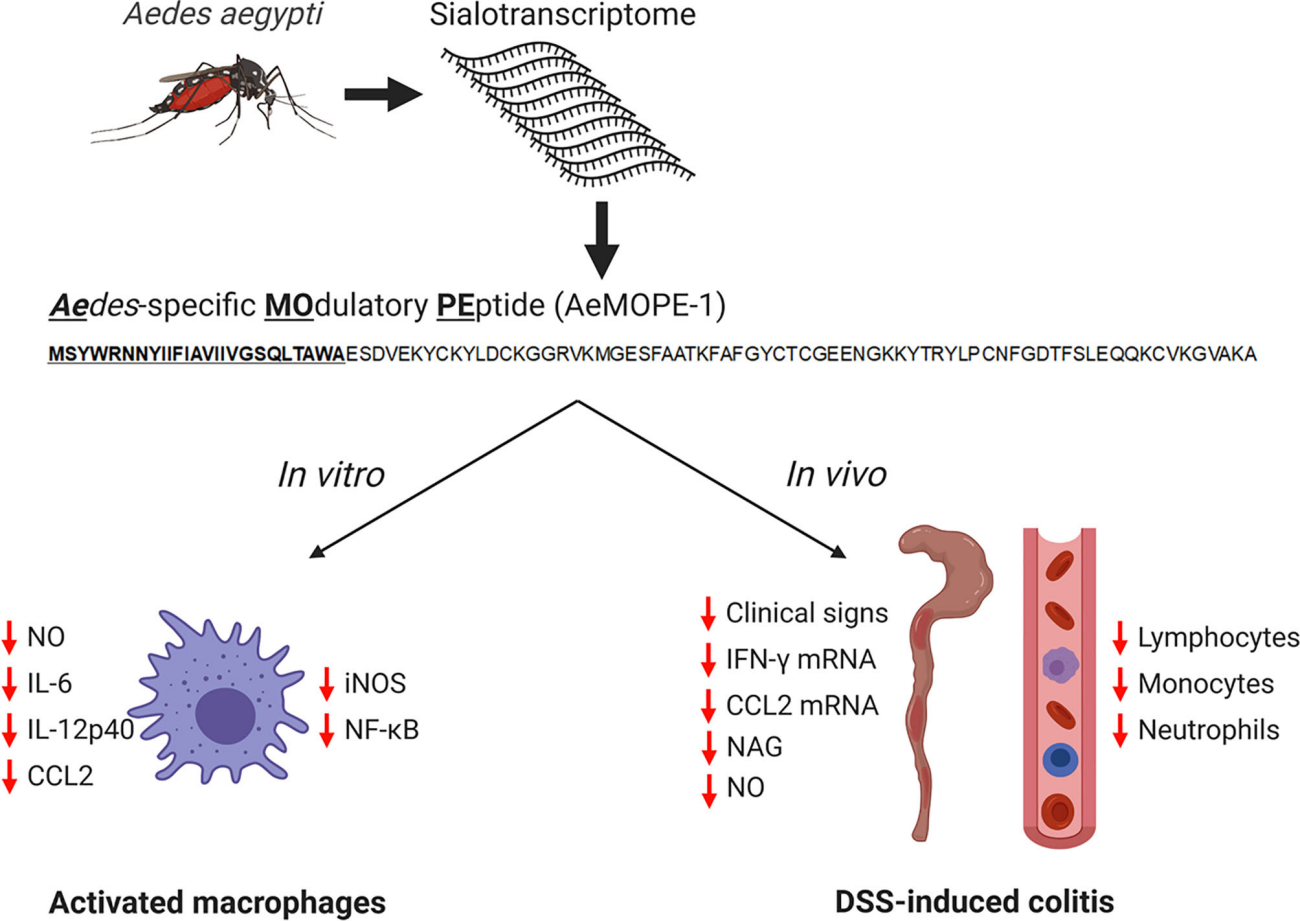
Immunomodulatory activities of syn-AeMOPE-1 *in vitro* and *in vivo*. The AeMOPE-1 transcript was identified in *Aedes aegypti* sialotranscriptomes. The mature secreted form of the peptide was synthetized and evaluated in murine macrophage cultures and in DSS-induced colitis. The immunomodulatory activities of the peptide are depicted in the figure. (Created with BioRender.com).

Together, our findings show that syn-AeMOPE-1 – a novel immunomodulator found in *Ae. aegypti* saliva – accounts for some of the *in vitro* and *in vivo* phenotypes previously reported for crude mosquito salivary preparations. The biological effects of syn-AeMOPE-1 uncovered by our work are illustrated in **Figure 7**. Thus, AeMOPE-1 joins an increasing list of immunomodulatory molecules present in *Ae. aegypti* saliva whose host target and mechanisms of action have been elucidated. In addition, this study highlights the importance of investigating the biochemical and pharmacological activities of *Ae. aegypti* saliva and salivary components, not only for understanding vector-host interactions, but also for the prospection of novel molecules with therapeutic properties and development of immunobiologics.

## Data Availability Statement

The raw data supporting the conclusions of this article will be made available by the authors, without undue reservation.

## Ethics Statement

The animal study was reviewed and approved by Ethics Committee on Animal Use (CEUA), under the protocol number 140/2011 (ICB/USP) and 11.1.1080.53.1 (FCFRP/USP). The procedures are according to the Brazilian National Law number 11794, which regulates the research activities involving animal use.

## Author Contributions

PL, JR, and AS-N conceived the study and designed the experiments. PL, EE, HS-C, JA, MH, MB, LN and AS-N performed experiments. PL, EE, HS-C, JA, JR, and AS-N analyzed data. PSJ, JM, CC, JR, and AS-N provided reagents/material/analytic tools. PL and AS-N wrote the first version of the manuscript. All authors contributed to the article and approved the submitted version.

## Funding

This work was supported by funds from the Fundação de Amparo à Pesquisa do Estado de São Paulo (FAPESP) grant # 2009/09892-6 (to AS-N), 2015/22934-0 (to AS-N), 2013/07467-1 (to PSJ) 2020/03175-0 (to JM) and 2013/00740-4 (to PL), Conselho Nacional de Desenvolvimento Científico e Tecnológico (CNPq) grant # 465678/2014-9 (to AS-N), 310174/2016-3 (to CC), 311204/2018-0 (to AS-N), 472744/2012-7 (to PSJ), 310993/2020-2 (to JM) and 309583/2019-5 (to CC), Núcleo de Pesquisa em Moléculas Bioativas de Artrópodes Vetores, University of Sao Paulo (NAP–MOBIARVE/USP) grant # 12.1.17661.1.7 (to AS-N), Coordenação de Aperfeiçoamento de Pessoal de Nível Superior (CAPES; Finance Code 001), and Intramural Research Program of the National Institute of Allergy and Infectious Diseases (Vector-Borne Diseases: Biology of Vector-Host Relationship) grant # Z01 AI000810-20 (to JR).

## Conflict of Interest

The authors declare that the research was conducted in the absence of any commercial or financial relationships that could be construed as a potential conflict of interest.
