# Supplementary material for: AeMOPE-1, a Novel Salivary Peptide From Aedes aegypti, Selectively Modulates Activation of Murine Macrophages and Ameliorates Experimental Colitis
Source: Front Immunol. 2021 Jul 19;12:681671. doi: 10.3389/fimmu.2021.681671 (PMC8327214; doi:10.3389/fimmu.2021.681671)
Supplement: Supplementary file 1 [file DataSheet_1.pdf]

Supplementary Figures

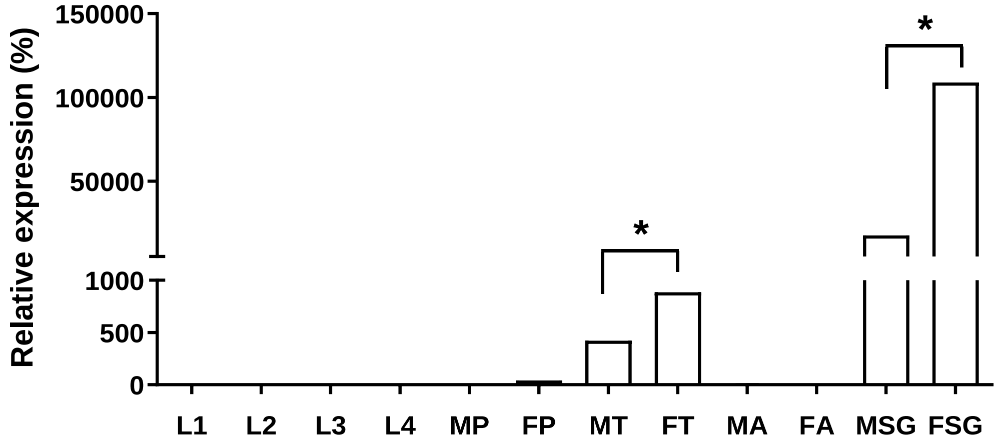

Supplementary Figure 1. AeMOPE-1 gene expression profile in different stages and tissues of *Ae. aegypti*. Gene expression was determined in larval stages L1 to L4 (L1-L4), male pupae (MP), female pupae (FP), male thorax (MT), female thorax (FT), male abdomen (MA), female abdomen (FA), male salivary gland (MSG) and female salivary gland (FSG) by RT-qPCR. Values are expressed as the mean ± SEM of $2^{-\Delta\Delta Ct}$ (n = 3). *$P \leq 0.05$ *versus* larval stage L1 - One-way ANOVA.

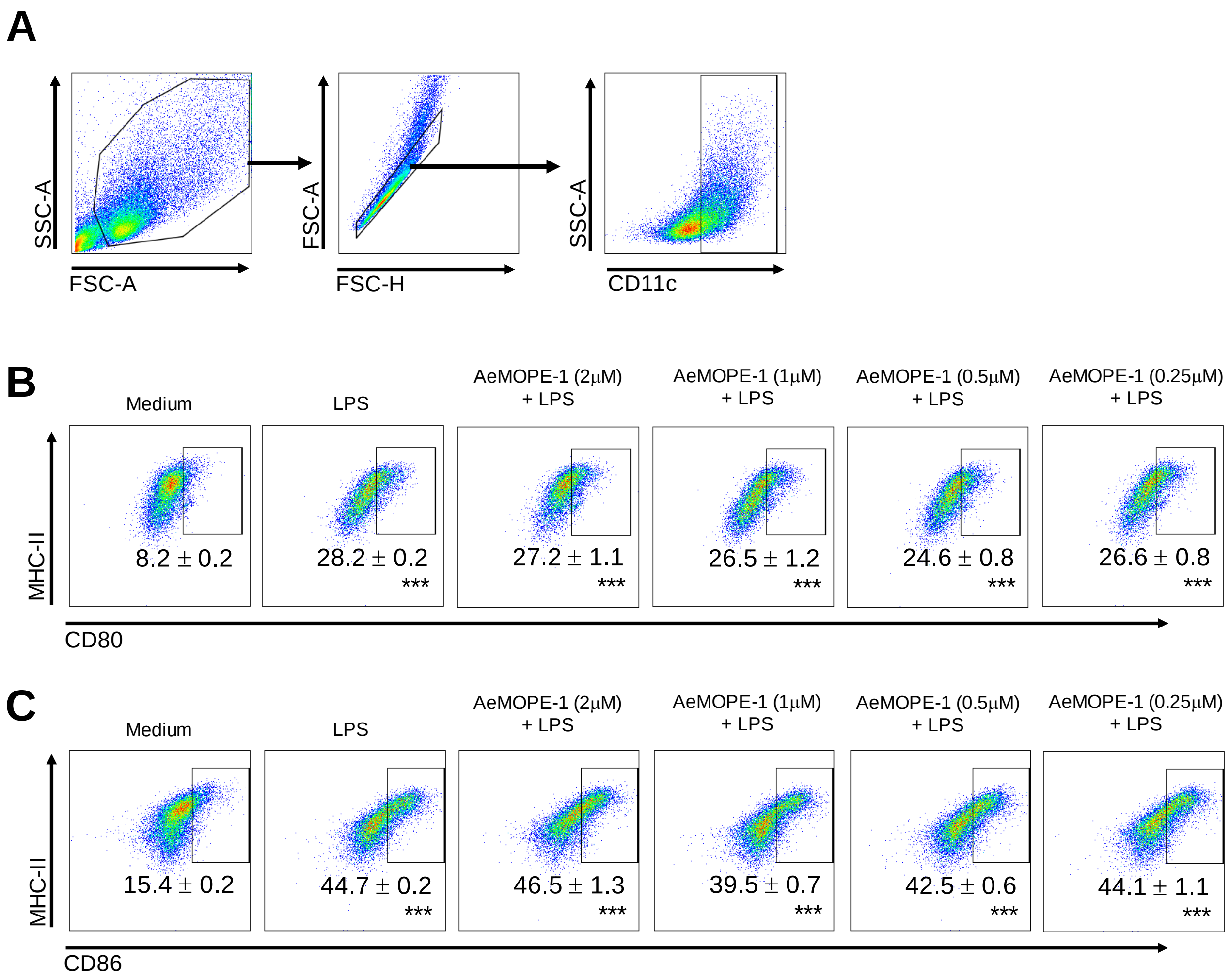

Supplementary Figure 2. Syn-AeMOPE-1 does not affect murine dendritic cell maturation. Bone marrow-derived dendritic cells were pre-incubated overnight with synthetic AeMOPE-1 (0.25 - 2 µM), stimulated with LPS (100 ng/mL) for 24 hours and the percentage of MHC class II^{+} CD80^{+} or MHC class II^{+} CD86^{+} cells were evaluated by flow cytometry. (A) Gating strategy. (B) CD80 expression in MHC class II^{+} cells. (C) CD86 expression in MHC class II^{+} cells. Results are expressed as the mean ± SEM from one representative experiment of four independent experiments assayed in quadruplicate. ***$P \leq 0.001$ *versus* unstimulated/untreated cells (Medium) – One-way ANOVA.

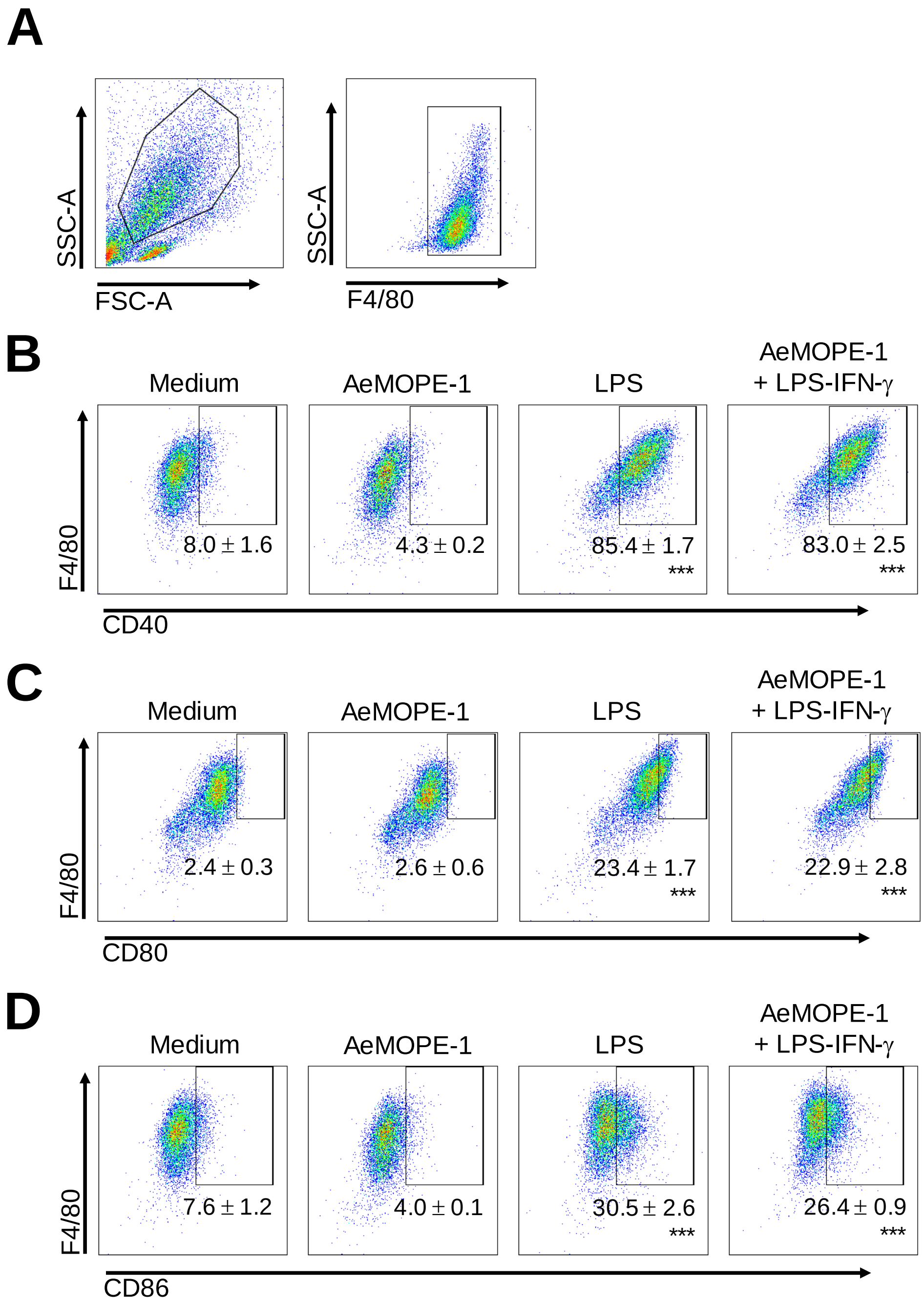

Supplementary Figure 3. Synthetic AeMOPE-1 does not affect the expression of accessory/costimulatory molecules by macrophages. Thioglycolate-elicited peritoneal macrophages were pre-incubated overnight with synthetic AeMOPE-1 (2 µM), LPS+IFN-γ (10 ng/mL of each) for 24 hours and the percentage of F4/80$^+$ CD40$^+$, F4/80$^+$ CD80$^+$ and F4/80$^+$ CD86$^+$ cells were evaluated by flow cytometry. (A) Gating strategy. (B) CD80 expression in MHC class II$^+$ cells. (C) CD86 expression in MHC class II$^+$ cells. Results are expressed as the mean ± SEM from one representative experiment of four independent experiments assayed in duplicate. ***$P \leq 0.001$ *versus* respective control group (Medium or AeMOPE-1) – One-way ANOVA.
